# The relationship between language and executive functions in adolescents with Down syndrome and fragile X syndrome

**DOI:** 10.1186/s11689-026-09697-x

**Published:** 2026-05-20

**Authors:** Audra Sterling, Marianne Elmquist, Amy Banasik, Brittany Ciullo, Tiffany Chavers Edgar, Jill Hoover

**Affiliations:** 1 Waisman Center, University of Wisconsin-Madison, Waisman Center, 1500 Highland Ave, Madison, WI 53705 USA; 2 Department of Communication Sciences and Disorders, University of Wisconsin-Madison, Madison, WI USA; 3 Department of Speech, Language, and Hearing Sciences, University of Massachusetts Amherst, Amherst, MA USA

**Keywords:** Intellectual disabilities, Executive functions, Language, Down syndrome, Fragile X syndrome

## Abstract

**Background:**

Individuals with fragile X syndrome (FXS) and Down syndrome (DS) have significant and pervasive challenges in language (and more specifically grammar) and executive functions (EFs). While these aspects of development are linked in autism and developmental language disorder, there has not been an investigation into this in FXS and DS. Thus, the purpose of this study was: 1) to evaluate the feasibility of experimental tasks for language and EFs, 2) to test if there are differences in language and EFs in DS and FXS, and 3) to test if EFs are related to grammatical abilities in DS and FXS within and between groups.

**Methods:**

Participants included 21 boys with FXS and 25 participants with DS (*n* = 9 females) between 9–17 years of age; groups were matched on chronological age (variance ratio = 1.13; *d* = 0.04, *p* = 0.897) and were similar on nonverbal IQ and vocabulary. Participants completed lab-based assessments including standardized assessments of nonverbal IQ and vocabulary, experimental measures of grammar (i.e., grammatical judgment and sentence imitation), three experimental executive function tasks, and a parent report of executive functions.

**Results:**

While there were participants who could not complete the tasks, overall the feasibility was high (72–91% participants completed the tasks). Wilcoxon rank-sum tests revealed no significant group differences in experimental grammar or EF tasks. In contrast, large differences emerged on parent-reported EFs, with greater impairment in FXS for shifting and inhibition. We used generalized linear regression models with Gaussian and binomial distributions to examine the relationships between EFs and grammatical abilities. We found that only working memory significantly predicted grammatical judgment.

**Conclusions:**

Participants with DS and FXS showed similar grammatical production and comprehension skills, contrasting with prior studies that relied on standardized testing and found more impaired production skills for children and adolescents with DS. Our sentence imitation task highlighted expressive grammar skills in DS, while grammaticality judgment posed challenges as a measure of grammar comprehension. Feasbility was good for all tasks, but there was a range, and younger participants in particular seemed to struggle with some of the tasks. The contrast in group differences between experimental and parent-reports of EFs calls into question whether the two measure EFs in a similar manner. Lastly, our study suggests that the language–EF relationships in intellectual disabilities may diverge from patterns documented in neurotypical development and language impairment without intellectual disability.

## Background

Fragile X syndrome (FXS) and Down syndrome (DS) are two of the most common genetic causes of intellectual disability. FXS is inherited, meaning it is passed along the X chromosome and results from an expansion on the *FMR1* gene [66], with a prevalence of 1 in every 7000 males or 11,000 females [30]; DS results from a third copy of the 21st chromosome and is not inherited, with a prevalence of approximately 1 in every 700 live births [38]. FXS and DS are both associated with intellectual disability, alongside language impairment [10, 17, 21]. There are many striking similarities between the two disorders: ear infections, higher rates of co-occurring autism, academic challenges, challenges with reading, and pervasive impairments within the language system [7, 9, 47, 63].

There are also, however, key differences between these two heterogeneous groups, including higher rates of autism in FXS, and differences in strengths and challenges within the language system [21, 25, 50, 51]. Due to the X-linked nature of FXS, males and females are impacted differently. Most males have intellectual disability and language impairment, whereas females with FXS have varying levels of intellectual and language abilities, ranging from impaired to within the neurotypical range [2, 3, 43, 59]. To control for the variability associated with sex-based differences in FXS and to reduce sex-based heterogeneity in the sample, this study and literature review focused on males with FXS. However, the findings are more mixed in DS regarding sex differences (e.g., [55], and there are not well documented language and EF differences between males and females with DS,thus we included both sexes.

By studying FXS and DS together, we have the unique opportunity to take a bottom-up approach to the study of language and cognition. Beginning with the known genetic disorder, we can then study the impact on cognition and language. This is particularly important in the case of two disorders with a clear genetic etiology. Given that they are both associated with intellectual disability, this allows us to examine the unique role of each genetic difference on development including, language and more specific aspects of cognition, namely executive functions (EFs), which have been linked in studies in autism, developmental language disorder, and neurotypical development [14, 15, 32].

EFs refer to a set of cognitive processes used to plan, manage, and control thoughts and behaviors [11]. EFs are often defined by three core areas: inhibition (i.e., inhibitory and/or behavioral control, ability to control one’s thoughts, behaviors, and/or emotions), working memory (e.g., actively holding and engaging information that is not readily present for temporary use), and shifting (e.g., ability to move from one task to another; shift attention) [11]. These skills are critical for adapting to one’s environment [41]. Studies have linked EFs to academic achievement and social development in neurotypical children and children with language impairments [6, 31, 32]. EFs have become an area of increasing interest in both DS and FXS, as they are sensitive to growth [27] and linked with key outcomes that are a struggle for individuals with FXS and DS including literacy and language [5, 19, 33, 72].

Language is multi-faceted and impacts nearly all aspects of daily life. Grammar, a core component of language, plays a critical role in academic success. Grammar supports reading and written language and facilitates conversations with peers by enabling individuals to express themselves and understand their communication partners. Grammatical production and comprehension are a challenge for individuals with DS and FXS, with more substantial challenges in production compared to comprehension in DS [21, 35]. For children with FXS, both grammatical production and comprehension are weaknesses, but the degree of impairment often depends on measurement type and comparison group [18, 58, 60]. Importantly, EFs and grammar are closely related [14]. In fact, the hierarchical competing systems model of language discusses in depth the importance of language for the development of EFs [39]. In this model, language allows children to reinforce representational concepts to override habits, since both the habit and representational system are needed to complete EF tasks [39, 74]. This model, however, was not developed with individuals with intellectual disability in mind, and thus it is unclear how language and EFs interact within development.

### EFs in DS and FXS

EFs have been systematically studied in DS and several studies have been completed in FXS, including both verbal and nonverbal EF tasks [26, 54–56, 68, 71]. In a recent meta-analysis, Tungate and Conners [65] considered EFs relative to mental age-matched neurotypical peers and the differences between verbal and nonverbal EF tasks. They included studies across a broad age range (mean age = 14 years,range: 6–40 years of age), and found that in general, EFs are a significant weakness for individuals with DS relative to mental age-matched neurotypical peers. They noted a unique profile for DS with working memory and shifting being more impaired than inhibition. They reported a particular challenge with verbal EF tasks, reinforcing the importance of considering verbal versus nonverbal tasks. Individual studies have considered the role of covariates in EFs and found varying results. One study evaluated verbal fluency in DS and found associations between IQ but not sex [55], while others have not reported assocations but controlled for cognition [56]. Floor effects are a concern in standardized assessments [16, 54], leaving open the question of alternative methods for examining EFs in school-age children.

Boys with FXS experience broad challenges with EFs. Schmitt and colleagues [53] completed a review on EFs and found that boys with FXS were, in general, below chronological age expectations on EF tasks including inhibitory control and working memory. Across studies, there were differences based on whether the tasks were verbal or nonverbal, with a similar trend identified in studies on DS, with more weakness in the verbal tasks [53]. A challenge in studies considering possible interactions between EFs and language involves the careful selection of EF tasks. Verbal EF tasks provide important information, but in populations with language impairment it is challenging to disentangle the language impairment from the EF construct of interest in a verbal-based task. Therefore, nonverbal EF tasks provide a promising alternative in FXS and DS in studies related to language and EFs.

### Grammar in DS and FXS

Grammar is essential for academic success and effective communication in social contexts, yet it is a particular weakness for individuals with DS. Loveall and colleagues completed an in-depth analysis of one specific aspect of grammar, verb production, in school-age children and adolescents with DS compared to neurotypical children and children with an intellectual disability [35]. They found that children with DS did not produce as many verbs during a narrative task compared to their peers,the diversity of the verbs produced however, was similar to their peers. They hypothesized that verb production most likely required significant effort in their grammatical system and, potentially given the challenges in EFs such as working memory and verbal short-term memory, led in part to these language difficulties. Boys with FXS also have significant impairments in grammatical skills, with studies reporting grammatical abilities below mental-age expectations [18, 20, 51]. We have found that boys with FXS have difficulty with verbs including, past tense and auxiliary “do”, with relative strengths in third person singular -s and copula and auxiliary “be” on a norm-referenced assessment and a sentence imitation task [58, 60]. There were, however, variations in performance based on the task, with participants at times showing better accuracy with the norm-referenced measure and at other times with the sentence imitation task [58].

Studies have compared DS and FXS in grammatical abilities. One study found that individuals with DS have challenges in expressive grammar relative to nonverbal mental age and in comparison, to other intellectual and developmental disabilities (IDDs) [21]. More specifically, adolescents with DS scored significantly lower than nonverbal mental age-matched peers with FXS and neurotypical peers on a standardized measure of expressive grammar, but there were no significant differences between the three groups on vocabulary production and comprehension. Martin and colleagues [40] also found that participants with DS scored significantly worse on a measure of syntax production compared to mental age-matched peers with FXS. Loveall and colleagues [37] completed an in-depth investigation of the different types of vocabulary children with DS understand (e.g., verbs, nouns or attributes/adjectives) compared to children with intellectual disabilities and neurotypical peers. Again, they found that children with DS had a weakness in verb comprehension compared to mental age-matched peers with intellectual disability.

Much of the literature to date in DS and FXS has focused on grammatical production. However, grammatical comprehension is also important to study as it is essential for academic success, in particular, reading comprehension [13, 46], yet very little has been done to understand this aspect of language in DS and FXS. Although grammatical comprehension is not yet well understood in DS and FXS, initial studies have found that both groups perform below mental-age expectations on standardized measures of language comprehension [44, 47].

Studies have used standardized tests [21, 58], and language samples to assess grammatical production [18]. However, language samples only allow for language production measures and are constrained by what the child spontaneously produces,standardized tests are prone to floor effects (e.g., [16, 54]). A review by Loveall and colleagues [36] evaluated 57 different standardized tests to assess language in children with IDDs. They found that although many reported having included individuals with IDDs in their norming sample, the sample sizes were limited. Additionally, only four of the 57 assessment tools had standard scores lower than 40, allowing for flexibility with floor effects [36]. Experimental measures offer a possible alternative to standardized tests and measures like sentence imitation have been used in FXS with good success [58]. In fact, in our own work, we included a sentence imitation task in school-age boys with FXS and found performance was not correlated with nonverbal IQ, and there was good convergent validity with a norm-referenced measure of grammar [58]. Experimental tasks have been used in autism and developmental language disorder with good success [14, 15, 24] and offer an alternative method for systematically evaluating language without the challenge of floor effects.

### Overview of the present study

Grammatical comprehension and production are both particularly challenging aspects of language for children and adolescents with DS and FXS. However, they are both important for critical aspects of daily life including academic success and literacy (e.g., [72]). Considering EFs and grammar together allows us to understand these complex inter-relationships and narrow our focus for potential intervention targets related to grammar and cognition. The ability to study these constructs in two intellectual and developmental disabilities with known genetic origins, like DS and FXS, provides a unique opportunity to consider the impact of genetics on language and cognition. It could be that there are unique differences between DS and FXS in grammar and/or EFs,or if the groups are similar, perhaps the challenges are more deeply related to intellectual disability and the general challenges noted in language for both groups. Importantly, given the challenges with standardized tests and floor effects, we first wanted to determine the feasibility of the experimental tasks before a deeper investigation into differences between groups on the tasks. Given the past literature, and this interest in disentangling the complex relationships between EFs and language, we had three research questions.

1. What is the feasiblilty of experimental tasks for DS and FXS for language (grammatical judgment and sentence imitation) and EF tasks (inhibition, shifting, and working memory)? Given that standardized tests are prone to floor effects, we opted to use experimental tasks. Our first step was to evaluate the feasibility of the tasks. Based on prior literature [14], we predicted that our participants would be able to complete the tasks
2. Are there differences between FXS and DS in grammatical abilities and EFs? We hypothesized that individuals with DS would have challenges in all aspects of grammatical abilities, and have poorer performance on the grammatical tasks compared to participants with FXS given past studies [21]; however we hypothesized the groups would have similar performance on EF tasks (e.g., [53, 65]).
3. How are EFs related to grammatical abilities in DS and FXS and does this differ across diagnostic groups? We hypothesized that grammar and EF performance would be related in both DS and FXS; given our hypothesis on poorer performance on grammatical tasks in DS we expected the relationships to be different between groups.

## Methods

Participants were drawn from a larger, longitudinal study on the relationship between language and EFs that included participants with FXS and DS (R01 DC19092); the participants with DS were seen at only one time point. Thus, we will report the findings from the first time point. This multi-site study included the University of Wisconsin-Madison and the University of Massachusetts Amherst. Participants included 21 boys with FXS and 25 participants with DS (*n* = 9 females) between the ages of 9–17. Participants had previous genetic testing to confirm their diagnosis. Participants were included in the larger study if they were monolingual English speakers (confirmed by parent report), passed a hearing screening, and communicated in at least 2–3-word phrases. Participants were excluded from the study if they were not using speech as their primary means of communication. We only included males with FXS given the documented sex differences in FXS (e.g., [2, 3, 43]). See Table 1 for participant demographics. Participants were recruited from research registries at the Waisman Center, outreach to the National Fragile X Foundation and DS-Connect, tabling events, emails to previous participant families, social media posts, flyers, and word-of-mouth.

**Table 1:** Participant demographics

Variable	DS (*n* = 25)	FXS (*n* = 21)	
	N (%) unless specified otherwise	N (%) unless specified otherwise	
Mean Age in years (SD)	13.33 (2.75)	13.47 (2.52)	
Sex			
Male	16 (64%)	21 (100%)	
Female	9 (36%)	0 (0%)	
Race			
Asian	2 (8%)	1 (5%)	
Black or African American	1 (4%)	1 (5%)	
White	20 (80%)	19 (90%)	
Unknown	2 (8%)		
Ethnicity			
Hispanic	2 (8%)	3 (14%)	
Non-Hispanic	20 (80%)	14 (67%)	
Unknown	3 (12%)	4 (19%)	
Family Income			
Less than $25,000		1 (5%)	
$25,000 – $50,000	1 (4%)	1 (5%)	
$50,000 – $75,000	2 (8%)	4 (19%)	
$75,000 – $100,000	4 (16%)	4 (19%)	
$100,000 – $150,000	7 (28%)	3 (14%)	
$150,000 – $250,000	6 (24%)	5 (24%)	
More than $250,000	4 (16%)	3 (14%)	
Unknown	1 (4%)		
EVT – Raw *M* (*SD*)	79.95 (19.35)		88.24 (16.67)
EVT – SS *M* (*SD*)	66.70 (9.38)		70.29 (10.32)
PPVT – Raw *M* (*SD*)	112.50 (34.56)		130.06 (34.88)
PPVT – SS *M* (*SD*)	55.68 (13.11)		66.12 (21.52)
Leiter-3 NVIQ SS *M* (*SD*)	50.59 (13.49)		46.44 (19.09)

*EVT* Expressive Vocabulary Test-Third Edition, *PPVT* Peabody Picture Vocabulary Test-Fifth Edition, *NVIQ* Leiter-3 test of nonverbal intelligence and cognitive abilities, *Raw* Raw Score, *SS* Standard Score

### Group matching

Following guidelines outlined by Kover et al. [34], groups were matched on chronological age (variance ratio = 1.13; *d* = 0.04, *p* = 0.897). The groups were not significantly different on nonverbal IQ (variance ratio = 0.50; *d* = 0.26, *p* = 0.445), vocabulary comprehension (variance ratio = 0.37; *d* = −0.59, *p* = 0.103), or vocabulary production (variance ratio = 0.83; *d* = −0.37, *p* = 0.275). We elected to match on chronological age, while also keeping nonverbal IQ and vocabulary at similar levels given our questions on understanding the language and cognitive profiles in adolescents with IDDs, in addition to precedent from other studies [28, 63, 70].

### Procedure

All testing was completed at either the Waisman Center at the University of Wisconsin-Madison or the University of Massachusetts Amherst during a single visit. The IRB approved all study procedures, and UW-Madison’s IRB served as the main point of contact. After obtaining informed consent from the legal guardian, we obtained oral assent from the participants. Language and cognitive standardized assessments and the experimental computer tasks were administered by trained research assistants including graduate student clinicians, an SLP clinical fellow, and licensed SLPs. All examiners were trained to deliver the tasks prior to data collection, and conversations regarding administration and scoring questions were ongoing. All assessments were both audio- and video-recorded for later coding and transcription. Participants were given breaks as needed; they were paid for their participation and travel was reimbursed.

### Standardized assessments

#### Nonverbal IQ

The Leiter International Performance Scale, Third Edition (Leiter-3; [52]), brief IQ composite is commonly used in research on children with intellectual disabilities. Participants completed the Sequential Order, Form Completion, Classification and Analogies, and Figure Ground subtests, which are the four subtests needed to generate a Brief IQ score.

#### Vocabulary comprehension and production

Vocabulary comprehension and production were measured using the Peabody Picture Vocabulary Test-Fifth Edition [12] (comprehension) and the Expressive Vocabulary Test-Third edition [73] (production) as descriptive measures of the participants to help provide a general context about their language abilities. Both raw and standard scores are reported in Table 1.

#### The behavior rating scale of executive function – second edition (BRIEF-2)

Caregivers completed the BRIEF-2 [22]. This measure provides insight into the impact of EF skills on daily functioning that would not be obtainable through the experimental EF tasks described below. We used t-scores for the following subscales in our analyses: working memory, inhibition, and shifting. Scores 65 and above are considered in the clinical range for impairment, whereby higher scores are equivalent to more impairment.

### Language experimental tasks

We had two experimental language tasks: one to assess grammatical comprehension and one to assess production. Stimuli for both tasks included 20 verbs embedded into 40 sentences featuring five tense markers: third person singular, past tense (regular and irregular), auxiliary “be”, copula “be”, and auxiliary “do”. The verbs were balanced on syllable shape, phonotactic probability, and neighborhood density, which are known to influence the accuracy of grammar production in prior studies [29]. For both tasks, the verbs were used at least twice, with a different tense marker for each use of a given verb. Across the sentences, we balanced agents and objects/complements, number of morphemes, words, and syllables.

#### Grammatical judgment

We used a grammaticality judgment task to measure grammatical comprehension. Participants were taught to judge whether a sentence sounds ‘good’ or ‘not so good’. Half of the sentences were grammatical, meaning one of the five categories of tense markers was used in a target appropriate manner (e.g., “the girl is shaking the toy”) and the other half of the sentences were ungrammatical, meaning the sentence contained an omission error (e.g., “the girl ___ shaking the toy”).

The grammaticality judgment task was presented using DirectRT software on a laptop. All sentences were presented via an external speaker placed on the table out of reach yet directly in front of the participant with the volume set to 50%. Participants indicated whether the sentence sounded *‘good’* by pointing to a picture of a smiley face and *‘not so good’* by pointing to a picture of a frown face. The test stimuli were presented only to participants who were able to complete the practice items. Four participants were not able to complete the practice items. Examiners transferred the participant’s response (i.e., “good” or “not so good”) for each sentence to the laptop and a paper form for scoring purposes.

Hit rates (H) and false alarms (F) were computed via DirectRT. A hit was defined as correctly identifying a grammatical sentence (i.e., participant saying “good” to a grammatically correct sentence). A false alarm was defined as incorrectly judging an ungrammatical sentence as ‘good’. (i.e., participant saying “good” to an ungrammatical sentence). Hit and false alarm rates were used to calculate sensitivity (*A’* i.e., A prime) and response bias (*B”* i.e., B prime). Sensitivity is an index of how sensitive a child was to errors in a particular condition. Scores are between 0 and 1 and typically range between 0.5 and 1. A score of 1 indicates perfect performance (i.e., 100% accuracy in both conditions), and scores of 0.5 indicate the participant could not discriminate between grammatical and ungrammatical sentences. Scores can fall below 0.5, which indicates response confusion. Response bias was used to measure a participant’s tendency to respond “good” or “not so good” to stimuli. Scores range from −1 to 1. A score of 0 indicates no response bias, a score of −1 indicates an extreme bias of responding *“good”,* and a score of 1 indicates an extreme bias to responding “*not so good*”. Sensitivity and response bias scores are used to assess performance on yes/no tasks like the grammatical judgment task (e.g., [15, 49]). Sensitivity and response bias scores were calculated following procedures outlined by Stanislaw and Todorov [57]. We opted to use these procedures because the procedures outlined in Grier [23] fail to provide a formula for calculating *A’* and *B”* when H < F (see [57] for more details on the history of using A’ and B” to assess performance on yes/no tasks like grammatical judgment). In cases (*n* = 3) where we had undefined values (i.e., extreme response bias leading to denominators being 0), we assigned that participant an *A’* score of 0.5 (i.e., not able to discriminate between grammatical and ungrammatical sentences).

#### Sentence imitation

We used a sentence imitation task to measure grammar production. Participants were asked to repeat verbatim 40 sentences, and their responses were audio-recorded. The 40 pre-recorded sentences featured five different verb tenses: third person singular (*n* = 10), Regular Past Tense (*n* = 5), Irregular Past Tense (*n* = 5), Auxiliary Be (*n* = 10), Copula Be (*n* = 10). The stimuli for the sentence imitation task were presented in the same way as described for the grammaticality judgment task. One demonstration sentence and three practice sentences were completed to check understanding prior to starting the task. The test stimuli were presented only to participants who were able to complete the practice items. Four participants (DS = 3, FXS = 1) did not complete the sentence imitation task.

Two trained research assistants transcribed participant responses verbatim offline using an audio file. Sentences were transcribed using the SALT conventions for coding bound morphemes, and omissions of bound morphemes were clearly marked. Word level codes in brackets were added to mark word level finiteness markers (e.g., production of the copula BE was coded as ‘is[COP]’). Both transcribers were required to pass a transcription training, which involved establishing reliability with gold standard coded transcriptions. Once transcribers passed their training, accuracy of all transcriptions was verified through a process of consensus coding where each transcriber independently transcribed and coded the responses from the task. Discrepancies were discussed, and a consensus was reached prior to scoring.

We applied a ‘target binary’ scoring system which focused on scoring the presence or absence of the finiteness markers. This method of scoring sentence imitation tasks has been used in prior studies, including in our own work [29, 69]. Applying the target binary scoring system required a two-step process: 1) determining whether a sentence can be scored, and 2) calculating the accuracy of the grammatical targets. To determine whether a sentence could be scored, the participant had to produce a fully intelligible subject that matched the subject number (i.e., singular or plural) or was the same as the subject provided in the stimulus. For third-person singular, past tense, and auxiliary BE sentences, the participant also needed to produce the main verb in the sentence. If these conditions were not met, the sentence was marked as ‘unscorable’. Across participants who completed the task, the mean for unscorable sentences was 0.34 (SD = 0.33; range: 0–1). Additional reasons sentences were determined unscorable included: subject-verb agreement errors (e.g., *the men cooks the food), a participant changing the target tense marker (e.g., after hearing a past tense sentence, the participant produced a sentence with the copula BE), or the participant not responding after the stimulus. Our “target-binary” score is intended to measure whether the child produced a specific finiteness marker presented in each target sentence. We therefore scored target finiteness markers as correct when they were produced and the child also correctly produced the original target subject (e.g., ‘The women are waiting for the train’. In contrast, we scored target finiteness markers as omitted if the child failed to produce the finiteness marker but retained the original subject that was presented in the stimulus (e.g., ‘The women ___waiting for the train’). Agreement errors (for example, ‘The *woman are waiting for the train’) are neither a clear omission nor a clear production of the target marker on the target subject. Treating those cases as “incorrect” would conflate a different error type with omissions and would change what the measure represents. For that reason, we set agreement errors aside as unscorable and report their frequency. This scoring system is in line with past research (e.g., [42]). All sentences determined to be unscorable were excluded from the accuracy calculation and all analyses. Once a sentence was determined to be scorable, accuracy of the grammatical target was marked as ‘correct’ or ‘incorrect’ if they omitted the grammatical target. We used an overall percent accuracy score. See Table 2 for more definitions.

**Table 2:** Definitions and examples for verb types targeted in sentence imitation task

Verb type	Definition	Example
Third person singular	Verb + present tense inflectional morpheme	“The boy **bites** the cookie”
Past Tense	Verb + regular past tense infelction morpheme OR production of irregular past tense form of verb	“The girl **pushed** the button” “The boy **fell** down the hill.”
Auxiliary “be”	is, am, are, was, were as the ‘helping’ verb	“The boys **are** waving to the train.”
Copula “be	is, am, are, was, were as the main verb	“The gray rabbit **is** hurt.”
Auxiliary “do”	do, does, did as the ‘helping’ verb	“**Does** the woman pour the milk?”

### EF experimental tasks

#### Working memory

Working memory was measured using a Corsi block task. Participants looked at an image on a computer screen that displayed an array of nine boxes. For each trial, the boxes lit up in an ordered sequence that the participant was immediately asked to replicate using a finger touch response. The task began with a sequence of two and stopped at a sequence of nine or when ceiling was reached. Each sequence level was repeated three times (e.g., three different trials of sequences of two). Ceiling was reached when a participant incorrectly repeated more than one of the trials at a given level. Participants were taught how to complete the task using three trials of a span of one. They were given feedback on the training trials, but not during the experimental trials. The full task was administered only for participants who completed these training trials. There were participants who passed the practice items but did not obtain a basal score (i.e., did not continue past level two); in these cases (*n* = 12), we assigned participants a score of 0. The dependent variable was the Corsi Block Total Capacity, or the longest sequence that the participant correctly repeated on at least two of three level trials. Scores can range from 0–9.

#### Inhibition

Inhibition was measured using a child-friendly flanker task. The participant was shown an image on a computer screen that consisted of a central directional object (e.g., a fish pointed in 1 direction) surrounded by objects on both sides (i.e., flankers). The flankers were either *congruent* with the central object (i.e., flanker fish pointed in the same directed as the central fish), *incongruent* with the central object (i.e., flanker fish pointed in the opposite direction as the central fish) or neutral (i.e., flankers surrounding the central fish were seaweed, nondirectional objects). Participants were provided teaching trials (*n* = 6) prior to the start of the task. There were 48 trials delivered via the Direct RT software (i.e., 24 congruent, 12 incongruent and 12 neutral). Participants went on to complete the 48 trials only if they were able to complete the training items. We used the incongruent accuracy score in analyses, which was the accuracy in the incongruent condition. We opted to measure accuracy rather than reaction time, in line with recommendations for individuals with an intellectual disability [26].

#### Shifting

Shifting was measured via the local–global task. The stimuli included 24 images where each image depicted one large shape comprised of several smaller shapes. We presented the images, one at a time, via a laptop computer running the Direct RT software. For half of the trials (*n* = 12), the participants were instructed to identify the global shape (i.e., a square); for the other half (*n* = 12), they were instructed to identify the local shape (i.e., the smaller shapes that make up the square). At each level (i.e., global and local), the shapes were congruent for four of the trials (small squares make up large square), incongruent for four of the trials (small circles make up large square), and neutral for four of the trials (small circles make up triangle). Participants were asked to touch the button that corresponds to the same shape at the level the participants were being asked to attend to (local or global). Eight practice trials were administered prior to starting the task. Participants only completed the local–global task if they were able to pass the training items. Accuracy rate of the incongruent condition was our dependent variable; given the recommendations by Hessl et al. [26], we did not use reaction time.

### Data analysis

All data analyses were performed in the R statistical computing environment (Version 4.2.0; [48]). To answer our research questions, we conducted a series of descriptive and inferential analyses.

#### Preliminary analyses

Initial data visualization/inspection showed that grammar and EF variables were not normally distributed. We opted for non-parametric analyses when normality of dependent variables was required (e.g., group comparisons). As part of our preliminary analyses, for each diagnostic group, we conducted a series of Wilcoxon rank-sum tests to examine if there were site differences in measures. We found no significant site differences across our measures. Given the wide age range and the use of raw scores for experimental grammar and EF measures, we also completed Spearmanrank-order correlations between chronological age and the grammar and EF variables separately for each diagnostic group. We found no significant correlations between study measures and chronological age (DS: ρ from −0.04 to 0.14; FXS ρ from −0.06 to 0.07).

During our preliminary analyses, we found that 13 participants (DS = 6, FXS = 7) who completed the grammatical judgment task had *A’* scores below 0.5, indicating that while they were able to complete the practice items, based on the *A’* score, they did not understand the task. For analyses including the A’ scores for the grammatical judgment task (i.e., research question 2), we opted to exclude these participants from analyses involving grammatical judgment, given that it is unclear whether there was a clinically meaningful difference in response confusion between an *A’* score of 0.49 and 0.01.

##### Missing data

During preliminary analyses, we inspected the data for missingness. Across our grammar and EF variables, we found missing data ranging from 5–33% across diagnostic groups, and in most cases, this was not due to time constraints or technological issues. Given our small sample size and the lack of recommendations for imputing data in small samples, we opted to handle missing data using pairwise deletion.

#### Planned analyses

For our first research question, we used descriptive statistics and Wilcoxon rank-sum tests to examine differences in age and NVIQ between participants who completed the experimental tasks and those who did not.

For our second research question, we conducted a series of multiple generalized linear regression analyses. For grammatical production, we treated the outcome (i.e., percent accuracy) as continuous and thus used a Gaussian distribution. Separate regression models were estimated for each EF variable, with grammatical production accuracy serving as the outcome variable, and EFs and diagnostic group as predictors. In the initial set of models, EFs and diagnostic group were entered as main effects to examine the relationship between EFs and grammatical abilities and to determine whether diagnostic group accounted for additional variance beyond EFs. In a subsequent set of analyses, we included EF X diagnosis interaction terms to test whether the associations between EFs and grammatical production varied by diagnostic group. Diagnostic plots from these models indicated mild heteroscedasticity. Given the small sample size and presence of mild heteroscedasticity, we employed heteroskedasticity-consistent standard errors (HC3) to enhance the robustness of our inferences. We reran our models using robust regression, which yielded comparable coefficient estimates; we report results from the generalized linear regression models with robust standard errors for grammatical production accuracy.

For grammatical judgment, we treated the outcome as binary (correct or incorrect response) and used a binomial distribution with a logit link function. Separate regression models were estimated for each EF variable via the lme4 package [4]. For this set of analyses, we used item-level grammatical judgment data and thus conducted multi-level logistic regression with grammatical judgment task accuracy as the outcome (i.e., correct or incorrect response). Grammaticality condition (i.e., grammatical items versus ungrammatical items) was added as a fixed effect in all models. To account for the non-independence of observations, we included random intercepts for both participants and stimulus items. We centered all EF variables before adding them to the model to facilitate the interpretation of interaction effects. As with grammatical production, in our initial set of models, EFs and diagnosis were added as main effects. In a subsequent set of analyses, we included EF x diagnosis interaction terms to test whether the association between EFs and grammatical judgment differed by diagnostic group. In a further set of exploratory analyses, for working memory and shifting, we also conducted three-way interaction models among EFs, diagnosis, and grammatical condition. For all models, fixed effects are reported as log-odds with associated z-statistics and 95% confidence intervals. Confidence intervals were derived using parametric bootstrapping (500 iterations). We also report odds ratios (ORs) to improve the interpretability of the results.

## Results

### RQ1: feasibility of tasks

We evaluated the overall feasibility of the language and EF tasks. Additionally, we wanted to take a more in-depth look at whether chronological age and NVIQ were associated with the missing data. We combined diagnostic groups for these analyses, given the lack of significant group differences on the experimental tasks and the relatively few participants who did not complete the tasks.

#### Grammatical judgment

Eighty percent of participants (*N* = 37; FXS *n* = 18, DS *n* = 19) completed the practice items and subsequently the grammatical judgment task. Of those participants, 24 (FXS = 11; DS = 13) performed at or above chance levels, and 13 (FXS = 7; DS = 6) performed below chance levels, indicating response confusion. Nine participants (FXS = 3; DS = 6) did not complete the task. Three of these participants completed some trials (range: 12–27) but not the full 40, and were therefore excluded from the prior analyses involving grammatical judgment. For two of the participants, the task was not administered due to difficulty attending to the task. The remaining four participants did not complete the training items and therefore could not progress to the task. Participants who did not complete the grammatical judgment task were significantly younger (*W* = 163.5, *p* = 0.026, *M* = 11.77 years, *SD* = 2.28; range: 9–15.75) than those with a sensitivity score of 0.5 or above (*M* = 14.03, *SD* = 2.43, range: 9.08–17.83), but not those with scores below 0.5 (*W* = 31.5, *p* = 0.077, *M* = 13.76, *SD* = 2.33, range: 9–16.17). There were no significant differences in NVIQ between the groups (χ^2^ (2) = 0.11, p = 0.947).

#### Sentence imitation

Ninety-one percent of participants completed the sentence imitation task (*N* = 41, DS *n* = 22, FXS *n* = 20). Four participants did not (DS = 3, FXS = 1). Three of these participants also did not complete the grammatical judgment task. Participants who did not complete the sentence imitation task were significantly younger than those who passed the training items and completed the task (*W* = 147.5, *p* = 0.014; did not complete: *M* = 10.36, *SD* = 1.48; did complete: *M* = 13.82, *SD* = 2.44). For three of the four participants, NVIQ was not obtained. Thus, we did not assess group differences in NVIQ.

#### Corsi

Seventy-two percent of participants completed the Corsi task (*N* = 33, FXS *n* = 13, DS *n* = 20). Of the 13 participants (DS = 5, FXS = 8) with missing Corsi data, 11 did not pass the practice items, while two did pass the practice items but did not have usable data (e.g., participants did not wait to press the squares). The two groups did not differ significantly in chronological age (*W* = 281.5, *p* = 0.105, *r* = 0.24) or NVIQ (*W* = 166, *p* = 0.399, *r* = 0.14).

#### Flanker

Eighty-nine percent of participants completed the flanker task (*N* = 41, DS *n* = 23, FXS *n* = 18). Five participants (DS = 2, FXS = 3) did not complete the task; for two, technical issues occurred, and for the remaining three, they were not able to attend to the task. There were no significant differences in chronological age for those who did and did not complete the task (*W* = 127, *p* = 0.397, *r* = 0.13). Of the five participants who did not complete the flanker, NVIQ was not obtained for four of them. Thus, we did not compare differences for NVIQ.

#### Local global

Seventy-three percent of participants completed the local global task (*N* = 34, DS *n* = 20, FXS *n* = 14). Thirteen did not completethe task (DS = 5. FXS = 7): three had technical issues, and 10 could not complete practice items (*n* = 3) or had significant attention challenges (*n* = 7). Participants who completed the local–global task and those who did not were similar in chronological age (*W* = 164.5, *p* = 0.329, *r* = 0.15) and NVIQ (*W* = 175, *p* = 0.115, *r* = 0.25).

### RQ2: are there differences between FXS and DS in grammatical abilities and EFs?

We conducted Wilcoxon rank-sum tests with Bonferroni corrections to examine group differences between participants with FXS and DS across grammar (i.e., grammatical judgment and sentence imitation) and EF domains (i.e., working memory, inhibition, and shifting). Although small-to-medium group differences were observed in the experimental grammar and EF measures, none reached statistical significance before or after correction. In contrast, parent-reported EFs revealed significant, large group differences in inhibition and shifting after Bonferroni corrections. Parents reported greater impairment in the FXS group for shifting (*r* = 0.70, *p* < 0.000) and inhibition (*r* = 0.51, *p* = 0.006; see Table 3 for full results). No significant group differences were found in the BRIEF-2 working memory subscale.

**Table 3:** Results from Wilcoxon rank-sum tests with Bonferroni corrections assessing group differences

Variable	FXS Median (IQR)	DS Median (IQR)	*W*	Effect size –* r*	unadjusted *p*-value	Bonferroni *p*-value
Grammatical Judgment – sensitivity (*A’*)	0.5 (0.21)	0.67 (0.23)	220	0.18	0.388	1.000
Grammatical Judgment – Bias (*B”*)	0 (0.36)	0 (0.14)	170	0.05	0.813	1.000
Grammatical Production accuracy	0.79 (0.30)	0.70 (0.21)	171	0.12	0.448	1.000
Working Memory						
Corsi	0 (2)	2.5 (2.5)	201.5	0.32	0.057	0.524
BRIEF-2 *t*-score	67 (6.5)	64 (12)	187.5	0.21	0.155	1.000
Inhibition						
Flanker	0.54 (0.36)	0.67 (0.5)	232.5	0.11	0.509	1.000
BRIEF-2 *t*-score	69 (13.75)	61 (11)	101.5	0.51	0.001	0.006
Shift						
Local Global	0.5 (0.22)	0.63 (0.16)	171.5	0.19	0.265	1.000
BRIEF-2 *t*-score	74 (13.75)	62 (14)	46	0.70	< 0.000	< 0.000

Sample sizes for group comparisons: Grammatical Judgment: FXS = 11, DS = 13; Grammatical Production Accuracy on Sentence Imitation Task: FXS = 20; DS = 22; Corsi: FXS = 13, DS = 20; Flanker: FXS = 18, DS = 23; Local Global: FXS = 14, DS = 20; all BRIEF-2 variables Working memory: FXS = 20, DS = 25

*BRIEF-2* Behavior Rating Inventory of Executive Function – Second Edition

### RQ3: how are EFs related to grammatical abilities in DS and FXS, and does this differ across diagnostic groups?

To address our third research question, we ran a series of multiple generalized linear regression models. Grammatical production was modeled as a continuous outcome (i.e., percent correct), and grammatical judgment was modeled as a binary outcome (i.e., correct vs. incorrect). We ran separate models for each grammar and EF outcome.

First, we ran a series of main-effects models to examine if EFs predicted grammatical abilities and if group explained additional variance beyond EFs. For grammatical production, we found that none of the experimental EF measures significantly predicted grammatical production accuracy (all *p-values* > 0.112), and diagnosis did not significantly explain any additional variance in grammatical performance beyond EFs (all *p-values* > 0.452; see Table 4).

**Table 4:** Results of generalized linear regression models (with robust SEs) examining executive functions and diagnosis as predictors of grammatical production

Predictor	B	SE	*t*	*p*
Model 1: Working Memory				
Corsi	0.05	0.03	1.59	0.112
Diagnosis: FXS	0.08	0.12	0.75	0.452
Model 2: Inhibition				
Flanker	0.18	0.14	1.30	0.195
Diagnosis	0.01	0.09	0.14	0.888
Model 3: Shifting				
Local Global	−0.03	0.31	−0.06	0.932
Diagnosis: FXS	−0.03	0.10	−0.318	0.751

Model 1: *n* = 32 (FXS = 13; DS = 19), χ^2^(2) = 0.21, *p* = 0.20, Pseudo-R^2^ (McFadden) = 1.06, AIC = 7.82. Model 2: *n* = 39 (FXS = 19; DS = 22), χ^2^(2) = 0.09, *p* = 0.472, Pseudo-R^2^ (McFadden) = 23.84, AIC = 6.48. Model 3: *n* = 33 (FXS = 14, DS = 19), χ^2^(2) = 0.01, *p* = 0.940, Pseudo-R^2^ (McFadden) = −0.07, AIC = 5.77

We ran a series of logistic mixed-level models to examine whether EF performance predicted item-level accuracy on the grammatical judgment task, controlling for diagnosis and grammaticality condition (e.g., grammatical versus ungrammatical items), and random intercepts for participants and item stimuli (See Table 5). For our experimental EF measures, only working memory (i.e., Corsi) significantly predicted greater accuracy on the grammatical judgment task (B = 0.12, SE = 0.06, z = 2.11, *p* = 0.035). Each one-unit increase in the Corsi was associated with a 13% increase in the odds of a correct response (OR 1.13). Across all grammatical judgment models, diagnosis was not a significant predictor of task accuracy (all *p-values* > 0.564). Grammaticality condition was a significant predictor across all models; ungrammatical items were associated with lower task accuracy (B = −0.89 to −1.17, all *p* < 0.001).

**Table 5:** Results of the logistic mixed-level regression models examining executive functions and diagnosis as predictors of grammatical judgement

Variable	B	SE	*z*	OR	95% CI	*p*-value
Model 1: Working Memory						
Fixed Effects						
Intercept	0.65	0.15	4.28	1.93	[1.42, 2.63]	< 0.001
Corsi	0.12	0.06	2.11	1.13	[1.00, 1.28]	0.035
Diagnosis: FXS	0.07	0.20	9.36	1.07	[0.71, 1.62]	0.716
Grammaticality: Ungrammatical	−0.90	0.15	−5.84	0.41	[0.30, 0.55]	< 0.001
Random Effects	Variance	SD				
Intercept – stimulus	0.08	0.28				
Intercept – Participant	0.11	0.33				
Model 2: Inhibition						
Fixed Effects						
Intercept	0.70	0.15	4.74	2.01	[1.51, 2.72]	< 0.001
Flanker	0.45	0.31	1.47	1.57	[0.84, 2.93]	0.143
Diagnosis: FXS	−0.06	0.17	−0.38	0.93	[0.67, 1.32]	0.703
Grammaticality: Ungrammatical	−0.89	0.16	−5.74	0.41	[0.30, 0.56]	< 0.001
Random Effects	Variance	SD				
Intercept – stimulus	0.11	0.33				
Intercept – Participant	0.12	0.35				
Model 3: Shifting						
Fixed Effects						
Intercept	0.86	0.17	5.11	2.36	[1.68, 3.34]	< 0.001
Local Global	0.02	0.63	0.03	1.02	[0.29, 3.54]	0.972
Diagnosis: FXS	−0.11	0.20	−0.58	0.89	[0.61, 1.30]	0.564
Grammaticality: Ungrammatical	−1.17	0.17	−6.85	0.31	[0.22, 0.44]	< 0.001
Random Effects	Variance	SD				
Intercept – stimulus	0.13	0.36				
Intercept – Participant	0.17	0.41				

Model 1: Observations = 1119, groups: stimulus = 40, participants = 28 (FXS = 13, DS = 17), AIC = 1481.2; Model 2: Observations 1399, groups: stimulus = 40, participants = 35 (FXS = 16, DS = 19), AIC = 1851.8. Model 3: Observations = 1199, groups: stimulus = 40, participants = 30 (FXS = 13, DS = 17), AIC = 1553.5

Given our hypotheses for our third research question, we examined whether the relationship between EFs and grammatical abilities differed between groups, even though diagnosis was not a significant predictor of task accuracy in any of our main-effects models. We included EFs and diagnosis as an interaction term. Given the results of our main-effects models and our sample size, these analyses should be interpreted as exploratory rather than confirmatory.

**Fig. 1:**
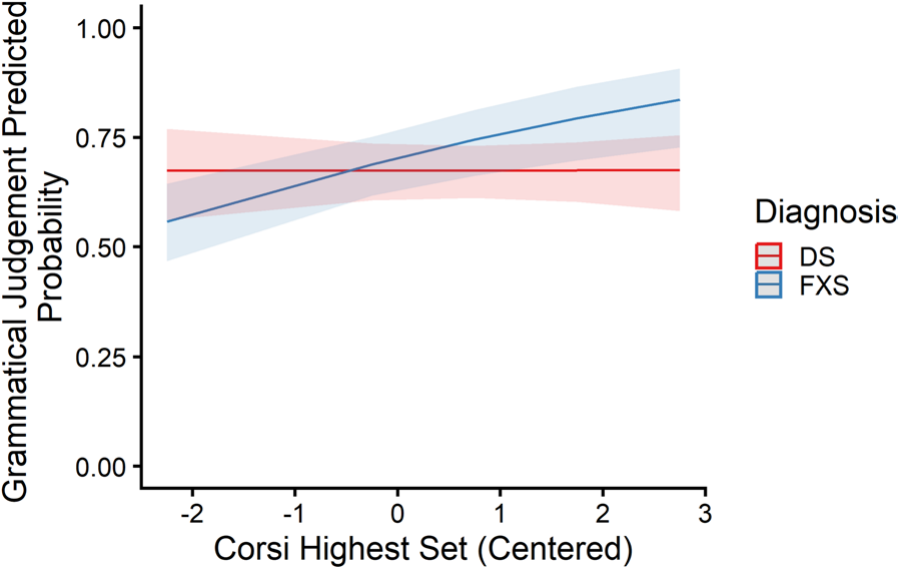
Predicted probability of correct response as a function of working memory

For grammatical production accuracy, we did not find a significant interaction between EFs and diagnosis (all *p-values* > 0.071; see Table 6). For grammatical judgment (See Table 7), we found significant interactions between EFs and diagnosis for working memory (B = 0.28, SE = 0.11, z = 2.60, *p* = 0.009) and shifting (B = 3.73, SE = 1.08, *z* = 3.46, *p* = 0.001). For working memory (see Fig. 1), the Corsi was not associated with grammatical judgment accuracy for the DS group (B = 0.00, SE = 0.07, z = 0.01, *p* = 0.991), whereas in the FXS group higher Corsi scores predicted greater accuracy on the grammatical judgment task, corresponding to 32% increase in odds of a correct response with each one-unit increase on the Corsi (OR = 1.32).

**Table 6:** Results of generalized linear regression models (with robust SEs) assessing interactive effects of diagnosis and executive functions on grammatical production

Predictor	B	SE	*t*	*p*
Model 1: Working Memory				
Corsi	0.05	0.04	1.16	0.246
Diagnosis: FXS	0.08	0.20	0.38	0.703
Corsi*Diagnosis	0.00	0.07	0.05	0.962
Model 2: Inhibition				
Flanker	−0.01	0.19	−0.06	0.954
Diagnosis	−0.28	0.20	−1.38	0.166
Flanker*Diagnosis	0.48	0.26	1.80	0.071
Model 3: Shifting				
Local Global	−0.25	0.37	−0.68	0.497
Diagnosis: FXS	−0.30	0.35	−0.85	0.395
Local Global*Diagnosis	0.47	0.63	0.74	0.460

Model 1: *n* = 32, χ^2^(3) = 0.21, *p* = 0.376, Pseudo-R^2^ (McFadden) = 1.06, AIC = 9.81. Model 2: *n* = 39, χ^2^(3) = 0.24, *p* = 0.242, Pseudo-R^2^ (McFadden) = 65.97, AIC = 5.66. Model 3: *n* = 33, χ^2^(3) = 0.05, *p* = 0.859, Pseudo-R^2^ (McFadden) = −0.41, AIC = 7.06

**Table 7:** Results of the logistic mixed-level regression models examining interactive effects of diagnosis and executive functions on grammatical judgement

Variable	B	SE	*z*	OR	95% CI	*p*-value
Model 1: Working Memory						
Fixed Effects						
Intercept	0.73	0.15	4.99	2.07	[1.54, 2.76]	< 0.001
Grammaticality: Ungrammatical	−0.90	0.15	−5.84	0.41	[0.29, 0.56]	< 0.001
Corsi	0.00	0.07	0.01	1.00	[0.87, 1.15]	0.991
Diagnosis: FXS	0.13	0.18	0.73	1.14	[0.77, 1.60]	0.465
Corsi*Diagnosis	0.28	0.11	2.60	1.32	[1.06, 1.65]	0.009
Random Effects						
Intercept – stimulus	0.07	0.27				
Intercept – Participant	0.07	0.26				
Model 2: Inhibition						
Fixed Effects						
Intercept	0.71	0.14	4.93	2.04	[1.53, 2.72]	< 0.001
Grammaticality: Ungrammatical	−0.89	0.16	−5.75	0.41	[0.31, 0.55]	< 0.001
Flanker	0.02	0.40	0.06	1.02	[0.49, 2.30]	0.953
Diagnosis: FXS	−0.05	0.16	−0.34	0.95	[0.67, 1.30]	0.733
Flanker*Diagnosis	0.95	0.60	1.59	2.58	[0.77, 7.49]	0.112
Random Effects						
Intercept –stimulus	0.11	0.33				
Intercept –Participant	0.11	0.33				
Model 3: Shifting						
Fixed Effects						
Intercept	0.89	0.15	5.82	2.44	[1.81, 3.31]	< 0.001
Grammaticality: Ungrammatical	−1.17	0.17	−6.86	0.31	[0.22, 0.44]	< 0.001
Local Global	−1.79	0.75	−2.40	0.17	[0.03, 0.75]	0.016
Diagnosis: FXS	−0.10	0.17	−0.59	0.91	[0.66, 1.29]	0.554
Local Global*Diagnosis	3.73	1.08	3.46	41.71	[4.20, 287.13]	0.001
Random Effects						
Intercept – stimulus	0.13	0.36				
Intercept – Participant	0.09	0.29				

Model 1: Observations = 1119, groups: stimulus = 40, participants = 28 (FXS = 13, DS = 17), AIC = 1477.0 Model 2: Observations 1399, groups: stimulus = 40, participants = 35 (FXS = 16, DS = 19), AIC = 1851.4. Model 3: Observations = 1199, groups: stimulus = 40, participants = 30 (FXS = 13, DS = 17), AIC = 1545.1

**Fig. 2:**
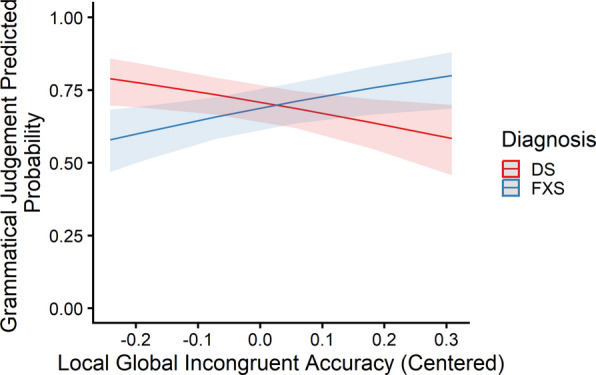
Predicted probability of correct response as a function of shifting

For shifting (see Fig. 2), we saw a cross-over interaction effect: in the DS group, higher shifting scores were associated with lower accuracy on the grammatical judgment task (B = −1.79, SE = 1.08, z = 3.46, *p* = 0.017). In contrast, in the FXS group, the association was positive (OR 6.97). Given the large interaction term (B = 3.73) and SE (1.08) and the cross-over nature of the effect, this effect should be interpreted cautiously. To aid interpretation of the significant interaction, predicted probabilities were calculated at ± 1 SD of the shifting variable. In the DS group, grammatical judgment accuracy decreased from 64% at lower local–global scores (−1 SD) to 51% at higher local–global scores (+ 1 SD). In contrast, in the FXS group, accuracy increased from 47 to 63% across the same range. As with the main-effects models, we continued to see that the grammaticality condition significantly predicted accuracy on the grammatical judgment task (B = −0.89 to −1.17, all *p-values* < 0.001).

In a further series of exploratory analyses, given the significant EF × diagnosis interactions on experimental measures of working memory and shifting, we examined whether these group-specific EF effects varied further as a function of grammaticality. We fit exploratory three-way interaction models (grammaticality × EF × diagnosis) to test whether the relations between EF and grammaticality judgment accuracy differed across item types within groups (See Table 8). Given the increased complexity of three-way interaction models and limited power to detect higher-order effects, these analyses are exploratory and interpreted cautiously.

**Table 8:** Results of the logistic mixed-level regression models examining interactive effects of diagnosis, executive functions, and grammatical condition on grammatical judgement

Variable	B	SE	*z*	OR	95% CI	*p*-value
Model 1: Working Memory						
Fixed Effects						
Intercept	0.66	0.17	3.80	1.94	[1.44, 2.82]	< 0.001
Grammaticality:Ungrammatical	−0.72	0.21	−3.38	0.49	[0.33, 0.73]	0.001
Corsi	0.47	0.10	4.51	1.60	[1.28, 1.98]	< 0.001
Diagnosis: FXS	−0.21	0.24	−0.85	0.81	[0.49, 1.31]	0.393
Grammaticality*Corsi	−0.88	0.12	−7.14	0.41	[0.32, 0.54]	< 0.001
Grammaticality*Diagnosis	0.64	0.30	2.15	1.89	[1.08, 3.58]	0.031
Corsi*Diagnosis	−0.46	0.15	−3.07	0.06	[0.45, 0.87]	0.002
Grammaticality*Corsi*Diagnosis	1.42	0.18	7.83	4.15	[2.89, 6.13]	< 0.001
Random Effects						
Intercept – stimulus	0.10	0.32				
Intercept – Participant	0.10	0.32				
Model 2: Shifting						
Fixed Effects						
Intercept	1.03	0.17	5.98	2.79	[2.04, 3.96]	< 0.001
Grammaticality: Ungrammatical	−1.45	0.21	−6.87	0.23	[0.15, 0.34]	< 0.001
Local Global	0.24	1.01	0.23	1.27	[0.16, 8.63]	0.816
Diagnosis: FXS	−0.34	0.23	1.50	0.71	[0.46, 1.07]	0.135
Grammaticality*Local Global	−3.99	1.20	−3.33	0.01	[0.00, 0.20]	0.001
Grammaticality*Diagnosis	0.55	0.26	2.09	1.73	[1.07, 2.88]	0.036
Local Global*Diagnosis	3.92	1.51	2.60	50.20	[2.17, 1358.52]	0.009
Grammaticality*Local Global*Diagnosis	−0.03	1.73	−0.02	0.97	[0.03, 22.77]	0.985
Random Effects						
Intercept – stimulus	0.15	0.38				
Intercept – Participant	0.11	0.32				

Model 1: Observations = 1119, groups: stimulus = 40, participants = 28 (FXS = 13, DS = 17), AIC = 1404.2 Model 2: Observations = 1199, groups: stimulus = 40, participants = 30 (FXS = 13, DS = 17), AIC = 1519.6

**Fig. 3:**
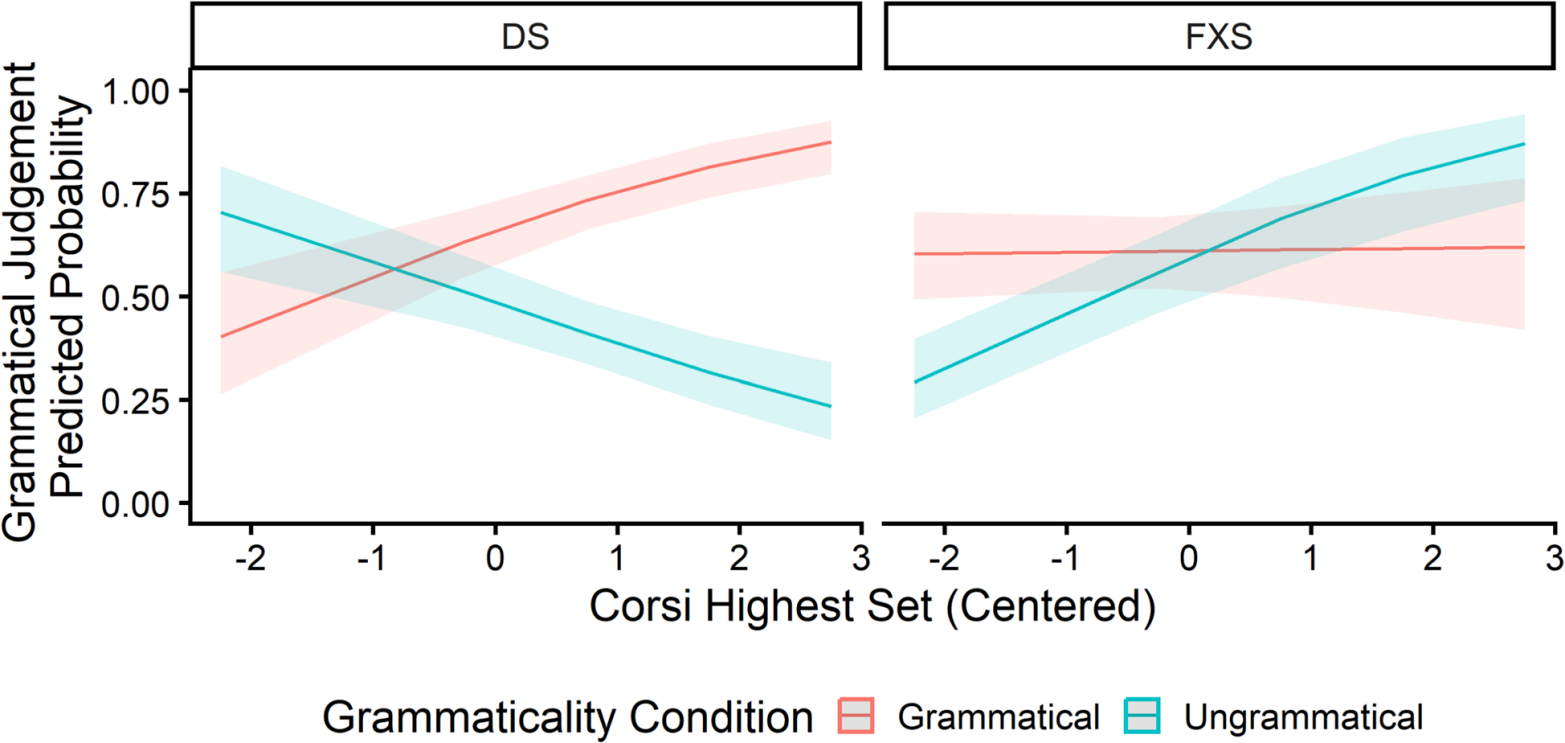
Predicted probability of correct response of working memory and grammar

For working memory (see Fig. 3), we found a significant three-way interaction between grammaticality, working memory, and diagnosis (B = 1.42, SE = 0.18, z = 7.83, *p* < 0.00), indicating that the relationship between working memory and grammatical judgment accuracy differed across diagnostic groups and varied by grammaticality (i.e., grammatical and ungrammatical items). To aid interpretation of the interaction effect, predicted probabilities were estimated at ± 1 SD of Corsi performance (−1.68, + 1.68). In the DS group, a cross-over pattern emerged for grammaticality. At lower Corsi performance (−1SD), accuracy was higher for ungrammatical items (65.4%) than for grammatical items (46.8%). However, at higher Corsi scores (+ 1SD), this pattern was reversed: accuracy increased for grammatical items (81%) and decreased for ungrammatical items (32.2%). In contrast, for the FXS group, for lower Corsi scores (−1SD), accuracy was higher for grammatical items (60.6%) than ungrammatical items (36.3). At higher Corsi scores (+ 1SD), ungrammatical accuracy increased to 78.8%, whereas accuracy for grammatical items remained relatively stable (61.7%).

For shifting, we did not see a significant 3-way interaction between grammaticality, shifting, and diagnosis (B = −0.03, SE = 1.73, *p* = 0.985), indicating that the relationship between shifting and grammatical judgment did not differ across grammatical and ungrammatical items.

## Discussion

We were interested in understanding similarities and differences in language and EFs in children and adolescents with DS and FXS. We used a combination of experimental tasks, parent report, and standardized tests to characterize our sample. Our first step was to examine the feasibility of the experimental tasks for language (grammatical judgment and sentence imitation) and EFs. Our completion rates ranged from 72 to 91%. Chronological age was associated with completion of the language tasks with younger participants struggling more to complete the task compared to older participants, but this was not true for the EF tasks. NVIQ was not associated with completion rate for any of the tasks. Notably, the language tasks were challenging; perhaps children at the younger age ranges do not have the grammatical skills necessary to complete the tasks. While our study provides preliminary data for these experimental tasks, it does offer an alternative for nonverbal EF tasks, which are important for studies examining the relationship between language and EFs. Additionally, it builds on prior literature, indicating success of these tasks and the relationships between EFs and language in other clinical groups, including autism and children with developmental language disorder [14, 24].

Part of the goal of this study was to examine alternatives to standardized testing, given the documented floor effects on standardized assessments [16]. While language samples can provide rich language production data [1, 62], they are limited to language production and what the individual spontaneously produces. As noted previously [61, 64], there are differences in findings based on the type of task. We have found this in our own work comparing standardized measures and experimental tasks for language measures [58]. This brings to light the critical importance of considering multiple assessment measures for both language and EFs for FXS and DS. Each assessment provides valuable information but perhaps measures slightly different constructs within the language and EF system. Future work should continue to disentangle this complex relationship and to better understand for whom and what purpose experimental tasks might be most appropriate.

Our second research question focused on group differences across the language and EF tasks; we found that participants were strikingly similar in their performance, and group differences did not emerge except for two domains (i.e., inhibition and shifting) from the parent-reported EF measure. In terms of grammaticality judgment, the groups were near chance (0.61 for FXS and 0.66 for DS). It is important to note that grammaticality judgment tasks are challenging in a way that other language comprehension tasks are not; they require a high level of metalinguistic awareness to judge the grammar of a sentence as ‘good’ versus ‘not so good’. In this way, our method of measuring grammatical comprehension may have been more complex than other measures of language comprehension commonly used in prior studies.

The participants had fairly high scores for sentence imitation (69% accurcy for FXS, and 68% for DS). Finestack and colleagues compared performance between participants with DS and FXS in a similar age group on standardized measures of grammatical production and comprehension [21]. They reported that the participants with FXS had significantly higher scores on the grammatical production measure compared to participants with DS, and the two groups were similar on comprehension. The difference in findings between our study and past studies is likely due to task differences. Finestack and colleagues used standardized assessments, while our study included experimental measures (e.g., [64]). Our experimental measures were specific to one aspect of grammar, finiteness marking on verbs, while the standardized tests measured a broader overview of the grammatical system. The sentences presented in our sentence imitation task were tightly controlled for sentence length with each sentence containing between five and seven words. We used a target binary scoring system (correct versus incorrect for the target morpheme only). While this is commonly used in experimental sentence imitation tasks and allows us to focus on finiteness marking, it does not capture other sentence-level production errors (e.g., semantic or other grammatical errors). These two features stand in contrast to sentence imitation tasks on standardized tests that typically feature sentences that are longer and more grammatically complex and utilize a more graded scoring system that captures more than accuracy of one specific grammatical marker. Thus, it is possible that the combination of length-controlled sentences and our target binary scoring method used for the accuracy calculation was not sensitive to detecting group differences. That said, the tightly controlled nature of our sentences allows us to demonstrate that grammatical finiteness marking may not, in fact, greatly differ between FXS and DS, despite prior studies pointing to potentially higher expressive grammatical skills in FXS.

It is noteworthy that our experimental measures of EFs did not result in significant differences between the groups, but the parent-reported measure did result in group differences for inhibition and shifting. Specifically, the parents of participants with FXS reported more impairment compared to the participants with DS, and in fact, the average score for the FXS group was in the clinically elevated range. This finding may represent the prior documented pattern that inhibition is a relative area of strength for individuals with DS according to experimental measures and the BRIEF-2 [8, 65], but it could also call into question the overlap and agreement between parent report measures of EFs and experimental measures. There have been investigations of this question in children without intellectual disability, including large samples of neurotypical children, and a broader review of 20 studies with child and adult participants [61, 64]. Studies consistently find differences, and Toplak and colleagues in their review argue that parent reported measures of EFs and more performance based measures seem to be measuring different underlying constructs in EFs [64]. While these issues have not yet been investigated in children and adolescents with intellectual disability, our findings call into question the possibility that this same argument might hold true for DS and FXS.

Our third research question asked how EFs were related to grammatical abilities in DS and FXS and whether there were differences across diagnostic groups. Most of our main-effects regression models were not significant, with one exception. Working memory predicted performance on the grammatical judgment task: specifically, greater working memory was associated with better performance. Across all our main-effects models, diagnostic group did not significantly explain additional variance beyond EFs. The grammaticality condition was a significant predictor in all of our multi-level logistic regression models examining grammatical judgment. Specifically, participants were more likely to misjudge ungrammatical items than grammatical items. We also ran exploratory models examining whether the relationship between EFs and grammaticality differed between FXS and DS. Given our modest sample size and SEs in some of our models, these findings should be interpreted with caution and warrant replication.

We did not find any significant interactions between diagnosis and EFs for grammatical production. However, for grammatical judgment we found significant interactions between EFs and diagnosis for working memory and shifting. Taken together, the working memory and shifting results suggest that there may be syndrome-specific differences in the relationship between EFs and morphosyntactic processing. For working memory, only the FXS group showed a positive association with grammatical judgment accuracy: with each unit increase on the Corsi substantially increasing the odds of a correct response on the grammatical judgment task. For the DS group, however, working memory was not related to grammatical judgment. The shifting results further reinforce this differentiation between the two groups. The cross-over interaction revealed that stronger shifting ability predicted higher accuracy on the grammatical judgement task in FXS, but lower accuracy in DS.

While this has not been investigated in DS and FXS to our knowledge, similar findings have been reported in other clinical groups. Ellis Weismer and colleagues [15] examined the role of nonverbal working memory in a grammatical judgment task similar to the task used in the current study in children with autism and children with developmental language disorder; they found that working memory predicted grammatical judgment performance across both groups. Similar findings have been reported for shifting. Ellis Weismer et al. [14] found that receptive language significantly predicted shifting in 8–12 year-olds with autism characterized by language impairment. Additionally, Peristeri and colleagues measured sentence processing and shifting in individuals with aphasia and found a similar relationship [45]. Given the lack of studies examining how EFs relate to grammatical judgment in DS and FXS, it is less clear why we saw a differentiation between groups. It could be that for individuals with DS, the role of online processing abilities like working memory do not play as strong of a role in either supporting order hinder performance during language tasks and instead may rely more heavily on long-term linguistic knowledge and implicit learning mechanisms. As some research suggests that implicit learning mechanisms in DS do not differ to mental-aged neurotypically developing children (e.g., [67]). Similarly, individuals with FXS may benefit from better shifting skills when judging morphosyntaic structures, whereas indvidiauls with DS may not deploy shifting in a way that supports the grammatical judgment task. If our findings are replicated, they would have implications for the development of language focused interventions for DS and FXS: EF-focused supports may be particularly important for FXS, whereas it may be have less of an added benefit for individuals with DS.

We also ran an exploratory three-way interaction between EFs, diagnosis and grammaticality condition for working memory and shifting. For working memory we found a significant three-way interaction between working memory, diagnosis, and grammaticality. For the DS group, better working memory facilitated correctly identifying grammatical items, but reduced the ability to reject ungrammatical items. Whereas in FXS, better working memory facilitated the ability to reject ungrammatical items, but performance on grammatical items remained relatively the same across working memory scores. The finding that working memory does not play a uniform role in morphosyntactic processing has been observed in another study that found the association between working memory detecting late versus early errors on a grammatical judgment task was reversed for children with and without language impairment [15]. Based on our sample size and large SEs, these findings should be interpreted cautsiouly and there is a need for future studies examining these relations further.

Studies like ours that document the feasibility of EF tasks lay the foundation for studies focused on testing interventions. For example, part of the excitement surrounding EFs in IDDs stems from work from Hessl and colleagues were able to report significant improvement in working memory for individuals with FXS after just 5–6 weeks of a working memory training program with some of the changes maintained at the three-month follow-up [27]. Additionally, teachers who were not aware of the training reported improvements in the classroom, indicating possible generalizability beyond the intervention context. While there is still work to be done to validate this training program and others, the data provide an exciting baseline to work from.

### Limitations and future directions

This study is not without its limitations. First and foremost, we had small sample sizes with relatively broad age ranges. While these age ranges are common in FXS and DS, they do require careful consideration as to how best interpret and apply the findings [18, 21, 28, 35, 58]. We controlled for sex differences in FXS by only including male participants, and thus our findings cannot be generalized to females. The small sample size and the large standard errors in some of our models indicate that our findings should be interpreted with caution and viewed as preliminary. Replication in larger samples that include both females and males with FXS will be important to further understand how EFs may support language development. We did not consider other additional, potentially important confounds including autism co-occurrence, and presence and severity of co-occurring ADHD. Systematically testing for autism and ADHD in both clinical populations, alongside previously documented medical information would allow for a more nuanced examination of this important set of variables. Both are common co-occurrences and might help explain some of the variance. Additionally, our sample included school-age and adolescent participants. Future work should continue to explore the relationships between EFs and language across the ages as it is possible that the nature of this relationship changes over the course of development as well as into the adult years. Understanding the developmental trajectory of language and EFs is crucial as both are skills essential for daily living across the lifespan.

## Conclusions

Our study comparing the language and EF skills of children with DS to children with FXS revealed several new insights. First, our study provides important documentation of the feasibility of nonverbal EF experimental tasks. Our study demonstrates that though some children experienced difficulty, the vast majority were able to complete the tasks supporting the use of nonverbal EF tasks in future studies. It will be important for future studies to tease apart the individual differences that contribute to successful task completion. Second, like previous studies, grammatical comprehension did not differ between FXS and DS, however, unlike prior studies, children with DS performed similarly to children with FXS on production of grammatical finiteness markers. The use of a sentence imitation task like ours that was length controlled and utilized a target binary scoring of finiteness markers revealed that children with DS may not have finiteness marking skills that are significantly weaker than individuals with FXS as previously documented via the use of standardized assessments. It is possible that standardized assessments with a higher working memory demand may have masked finiteness marking in DS. Moreover, we identified potential challenges associated with measuring language comprehension through the grammaticality judgment task which heavily relies on metalinguistic skills in a way that many comprehension measures do not (e.g., picture pointing tasks). Future studies may need to think carefully about the use of grammaticality judgment tasks as a measure of receptive grammar in intellectual disabilities. Lastly, the current study takes an initial step in extending the hierarchical competing systems model of language and EFs to individuals with intellectual disabilities [39]. We found that working memory predicted performance on the grammatical judgment task. Better working memory was associated with better performance on the grammatical judgment task. Diagnostic group did not significantly explain additional variance beyond EFs. We did not find any significant interactions between diagnosis and EFs for grammatical production. However, for grammatical judgment, we found significant interactions between EFs and diagnosis for working memory and shifting, a finding that could and should be expanded on in future studies. While the hierarchical competing systems model provided a framework to motivate the current work, our results indicate that the relationship in intellectual disabilities may diverge from what is known for neurotypical development and language impairment not associated with intellectual disability.

## Data Availability

The datasets generated and/or analyzed during the current study are available from the corresponding author on reasonable request.

## Abbreviations

ID: Intellectual disability
FXS: Fragile X syndrome
DS: Down syndrome
EFs: Executive functions
